# Visual recovery after perinatal stroke evidenced by functional and diffusion MRI: case report

**DOI:** 10.1186/1471-2377-5-17

**Published:** 2005-09-26

**Authors:** Mohamed L Seghier, François Lazeyras, Slava Zimine, Sonja Saudan-Frei, Avinoam B Safran, Petra S Huppi

**Affiliations:** 1 Department of Radiology, Geneva University Hospitals, Micheli-du-Crest 24, 1211 Geneva, Switzerland; 2 Laboratory for Neurology and Imaging of Cognition, Departments of Neurosciences, University of Geneva, Michel-Servet 1, Geneva 1211, Switzerland; 3 Department of Anesthesiology, Geneva University Hospitals, Micheli-du-Crest 24, 1211 Geneva, Switzerland; 4 Ophthalmology Clinic, Department of Clinical Neurosciences and Dermatology, Geneva University Hospitals, Geneva, Switzerland; 5 Department of Neurology, Children's Hospital, Harvard Medical School, Boston, USA; 6 Department of Pediatrics, Children's Hospital of Geneva, 6 rue Willy-Donzé, 1211 Geneva, Switzerland

## Abstract

**Background:**

After perinatal brain injury, clinico-anatomic correlations of functional deficits and brain plasticity remain difficult to evaluate clinically in the young infant. Thus, new non-invasive methods capable of early functional diagnosis are needed in young infants.

**Case Presentation:**

The visual system recovery in an infant with perinatal stroke is assessed by combining diffusion tensor imaging (DTI) and event-related functional MRI (ER-fMRI). All experiments were done at 1.5T. A first DTI experiment was performed at 12 months of age. At 20 months of age, a second DTI experiment was performed and combined with an ER-fMRI experiment with visual stimuli (2 Hz visual flash). At 20 months of age, ER-fMRI showed significant negative activation in the visual cortex of the injured left hemisphere that was not previously observed in the same infant. DTI maps suggest recovery of the optic radiation in the vicinity of the lesion. Optic radiations in the injured hemisphere are more prominent in DTI at 20 months of age than in DTI at 12 months of age.

**Conclusion:**

Our data indicate that functional cortical recovery is supported by structural modifications that concern major pathways of the visual system. These neuroimaging findings might contribute to elaborate a pertinent strategy in terms of diagnosis and rehabilitation.

## Background

After perinatal brain injury, early assessment of clinico-anatomic correlations is invaluable for the understanding of functional deficits and brain plasticity. Cortical visual impairment (CVI), defined as a visual deficit caused by a disturbance of the posterior visual pathways, remains difficult to assess clinically in the young infant [1]. In this context, new non-invasive methods capable of early functional diagnosis are needed in young infants. Functional mapping with magnetic resonance imaging (fMRI) was successfully performed in infants presenting alterations in the visual system [2,3]. The use of diffusion tensor imaging (DTI) was also demonstrated in young infants for the assessment of structural integrity [4,5]. The combination of both techniques (fMRI and DTI) has opened up the opportunity to investigate structure-function relationships non-invasively in children [6,7].

Cerebral reorganization following lesions in the visual pathways has been documented using a variety of neuroimaging techniques. [8-12]. Generally, studies have compared the activated cortical regions in damaged-brain subjects with healthy matched-control subjects in order to characterize the atypically activated regions as major indices of brain plasticity. An additional, and probably more interesting, approach consists of longitudinal functional investigations conducted in the same subjects to identify reorganization processes after initial lesion. Typically, these longitudinal studies have been extensively applied to adult subjects with structural [13,14] and functional [15-17] approaches. However, longitudinal studies have been performed in only few pediatric studies using visual evoked potentials [18,19], but none with functional neuroimaging. Furthermore, in previous functional neuroimaging studies, brain recovery was mostly described by visualization of functional activation with little neuroimaging evidence about underlying structural modifications and altered connectivity that concur with functional reorganization (e.g. [20-22]).

Previously, we have demonstrated the feasibility of combining fMRI and DTI in a very young infant [7]. In this study, we used such a combination to monitor the structural-functional correlates of the visual system recovery in an infant with a prominent left hemispheric lesion involving the temporo-parieto-occipital regions. Our data show that functional recovery is supported by structural modifications that concern the major optical tracts of the visual system.

## Case presentation

**Figure 1:**
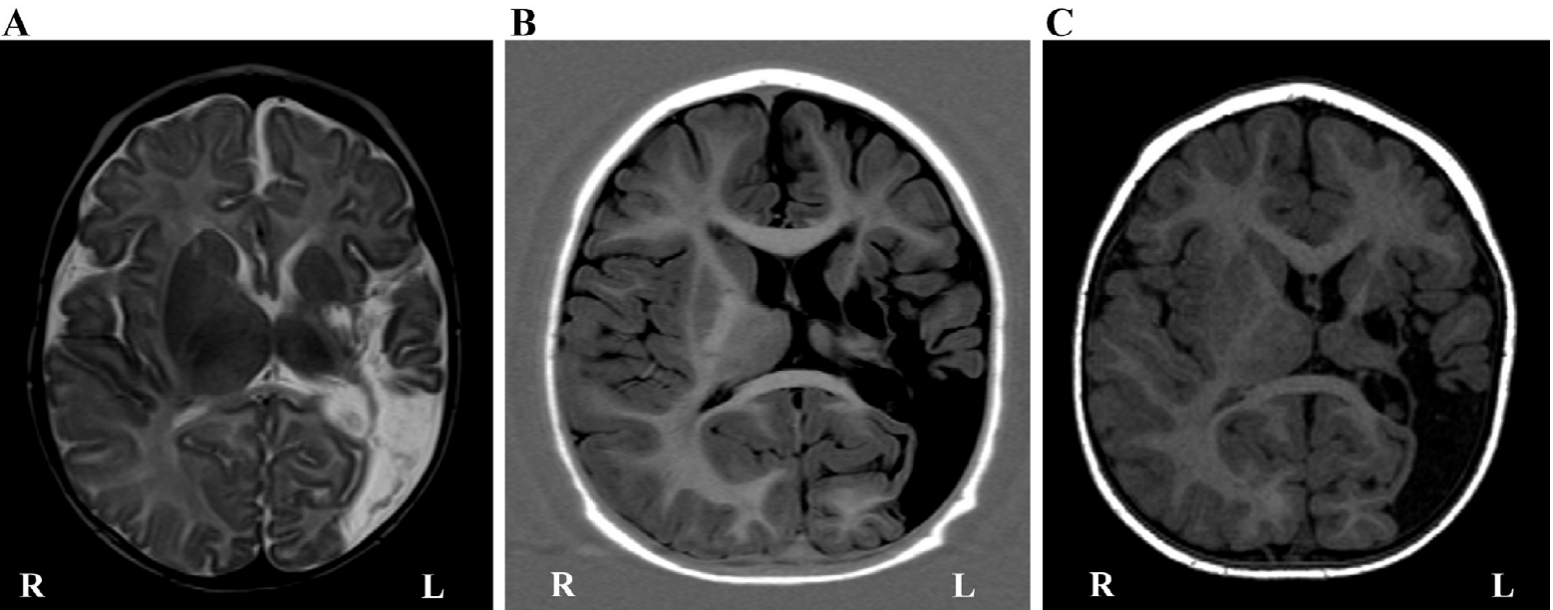
Routine MRI showing the extent of the lesion in the left hemisphere on an axial view, at (**A**) 3 months of age with T2-weighted image, (**B**) 12 months of age with an inversion recovery T1-weighted image, and (**C**) 20 months of age with a T1-weighted gradient-echo image.

An infant boy, born at 37 weeks of gestation, developed apneic episodes and focal seizures on the third day of life. Diffusion-weighted images at four days of age showed a prominent left hemispheric lesion, involving particularly the temporo-parieto-occipital regions, evolving into tissue dissolution and local atrophy. The extent of the lesion in the left hemisphere at different ages is shown in Figure 1. As clinical evaluation of cortical visual function was difficult to carry out in an infant, a combined fMRI-DTI evaluation was performed at 3 months of age to assess structural and functional integrity of the visual system. Visually stimulated cortical activation with fMRI was absent in the left hemisphere and the DTI showed absence of fiber tracts in the left occipital lobe (for more details see [7]). To assess the potential of visual recovery mechanisms in the left occipital lobe, an additional DTI evaluation was performed at 12 months and an fMRI-DTI combination was conducted at 20 months of age.

This study was approved by the ethical committee of the University Hospitals of Geneva. All experiments were performed on a 1.5T INTERA system (Philips Medical Systems, Best, Netherlands). A vacuum pillow (PAR Scientific A/S, Denmark) was used to minimize head movement.

### DTI methods

A SE-EPI sequence (b-factor = 700 s/mm^2^, TE = 65 ms) was used with 6 non-collinear gradient directions plus one non-diffusion-weighted B0 image [23]. 22 contiguous 4 mm slices (in-plane resolution = 1.56 × 1.56 mm^2^) were acquired during the DTI experiment at 12 months of age (noted as DTI-12), and 32 contiguous 3 mm slices (in-plane resolution = 1.95 × 1.95 mm^2^) were acquired during the DTI experiment at 20 months of age (noted as DTI-20). The acquisition was repeated four times to improve the signal-to-noise ratio. Diffusion was measured in terms of the apparent-diffusion coefficient (ADC) according to the Stejskal and Tanner equation. The matrix describing the directional dependence of the ADC was estimated for each voxel [24]. For each estimate, the three orthogonal eigenvectors and their related positive eigenvalues were calculated. For each voxel, a fractional anisotropy (FA) index, ranging from 0 to 1, was calculated according to Basser and Pierpaoli's definition [25]. The principal direction of diffusion is given by the eigenvector corresponding to the largest eigenvalue and represents the fiber bundle direction. White matter tracts were reconstructed with the streamline-like approach [26], with anisotropy threshold of 0.27 and angles less than 40° [27].

### FMRI methods

During the fMRI examination, conducted in the same session as DTI-20, the infant was sedated with Propofolum (8 mg/kg). Functional imaging consisted of a GRE-EPI sequence (TR/TE/Flip = 1 s/40 ms/80°, resolution 1.95 × 1.95 mm^2^, 12 contiguous 5 mm axial slices). An event-related approach was used, with stimulus (2 Hz visual flash) duration of 5 sec repeated 15 times with an inter-stimulus interval of 30 sec. Flashing frequency of 2 Hz was used as it yields robust visual evoked potentials in young infants (e.g. [28]). Visual stimuli were presented to the infant via a video projector, a front-projection screen and a system of mirrors fastened to the head coil. Throughout the examination, the eyes of the infant were closed. Data, after motion correction and spatial smoothing, were analyzed using SPM99 software package. All activations at p < 0.001 (uncorrected) are reported. In addition, the transformation matrix to co-register the mean functional image and the non-diffusion-weighted B0 image was calculated. This transformation matrix was then subsequently applied to the statistical maps in order to have the same space for both fMRI and fractional anisotropy (FA) maps.

## Results

**Figure 2:**
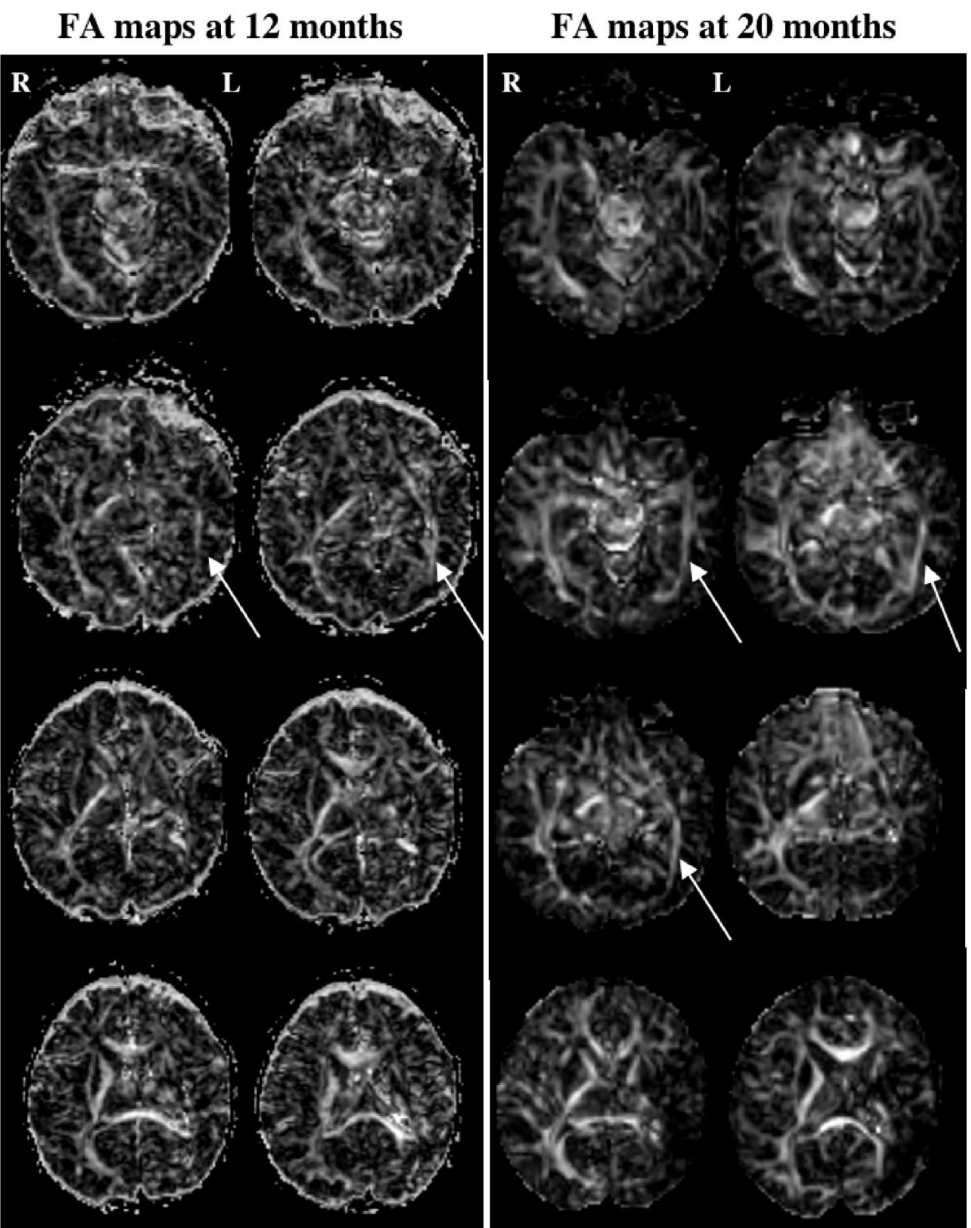
Fractional anisotropy maps presented with axial slices for the diffusion experiment at 12 months of age (left columns) and 20 months of age (right columns). The optic radiations in the left (injured) hemisphere are indicated by white arrows.

In both DTI and fMRI experiments, head movement was minimal leading to good quality images. The current results of these longitudinal experiments showed different new findings from the first fMRI-DTI combination at 3 months of age. Figure 2 illustrates the FA maps for DTI-12 and DTI-20 experiments. Optic radiations in the intact right hemisphere are visible in both experiments with comparable anisotropy values. In the injured left hemisphere, optic radiations are more prominent in DTI-20 than in DTI-12 experiment. The reinforcement of fibers in the left hemisphere is particularly visible in the antero-posterior direction.

**Figure 3:**
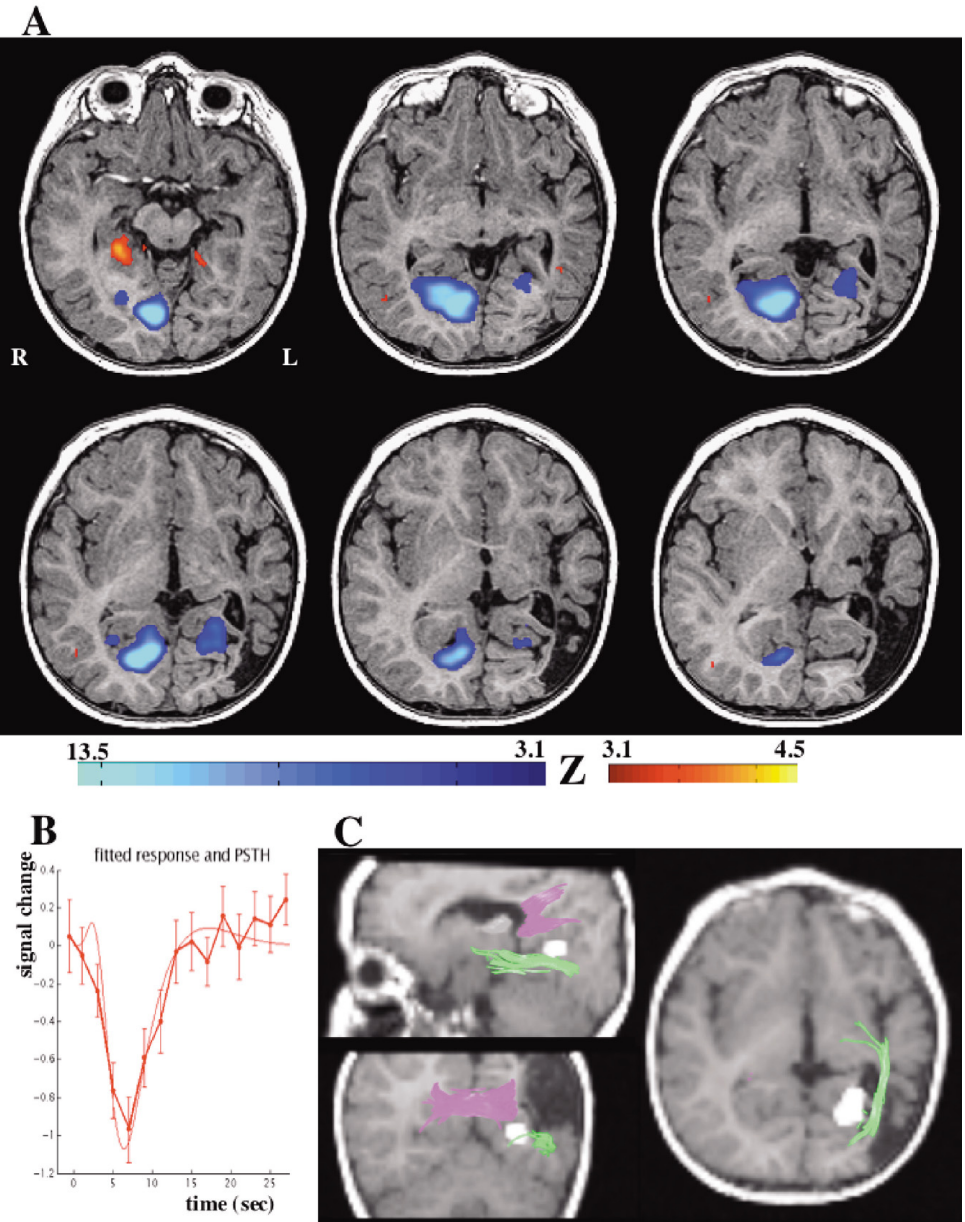
(**A**) fMRI statistically significant areas of activation (at p < 0.001, uncorrected) were projected on axial slices of the anatomical volume of the infant. Positive activation is shown in red colors and negative activation is shown with blue colors. The strongest activation is negative, in the occipital lobe. Note the activation in the left (injured) visual hemisphere. (**B**) The shape of the event-related hemodynamic response of the left visual cortex activation. (**C**) Correspondence between the DTI tracts and the fMRI activation in the left hemisphere is illustrated on three views. The functional activation is indicated with a white cluster superimposed on the anatomical volume of the infant. The optic radiation is shown with green color and the inter-hemispheric connections with pink color.

Activations in the ER-fMRI experiment in the visual cortex have essentially appeared with negative event-related responses (figure 3B). The strongest activation was found in the right (intact) occipital lobe (size = 350 voxels, T = 13.5, p < 10^-10 ^corrected), particularly in the anterior part of the striate cortex (i.e. V1 area). Surprisingly, a significant activation was also observed in the left injured hemisphere (size = 72 voxels, T = 6.1, p = 0.00002 corrected), located slightly more laterally and more anterior than visually activated areas in the right hemisphere (figure 3A). This left visual activation was located in the lingual gyrus (Brodmann areas 18/19), corresponding presumably to visual area V2. Moreover, the site of the left visual activation was compared to the white matter tracts obtained during the DTI-20. Figure 3C shows that the cortical activity lays below the inter-hemispheric fiber tracts from the forceps major and close to the ipsilateral optic radiation fibers

## Conclusion

At 3 months of age this infant with left-sided perinatal stroke, had shown activation upon visual stimulation only in the right intact hemisphere with both block and event-related fMRI paradigms [7]. At 20 month of age, functional maps with ER-fMRI showed again strong negative BOLD response in the right intact hemisphere, implicating principally the anterior part of the right primary visual cortex (V1). Such anterior negative responses were also reported previously in infants [2,29]. In addition, ER-fMRI maps at 20 months of age showed responses in the left injured hemisphere that were not previously observed. Before discussing the physiological basis of these functional modifications, some methodological aspects should be evoked. The critical issue inherent to any functional longitudinal studies is the test-retest reliability or the inter-session variability (see [30,31]). Recently, it was shown that the inter-session variability is not necessarily high and its magnitude is similar to within-session variability [32]. Also, the reproducibility of visual areas in fMRI is very high, even in higher-level visual areas [33]. Furthermore, to minimize inter-session variability, we have initially used both event-related and block paradigms at 3 months of age. The block paradigms were repeated three times and have shown comparable results with the ER-fMRI paradigm, indicating that the absence of significant activation in the left injured hemisphere could not be attributed to inter-session variability only [7]. At 20 months of age, we have also used block paradigms (data not shown here) and all results of block and ER-fMRI paradigms confirmed the detection of a significant activation in the left injured hemisphere. Further, the implication of the right hemisphere could be considered as a "witness" of the weak influence of inter-session variability, because this right visual cortex activation was present across sessions with comparable statistical significance. The modifications between functional maps at 3 months and 20 months of age could therefore illustrate functional recovery by plasticity and resilience of the developing brain.

Functional recovery observed in the left occipital lobe was supported by structural modifications evidenced in both DTI-12 and DTI-20 experiments. DTI-20 delineated fibers in continuity with the optic radiations and immediately adjacent to the cystic lesion. Thus, our DTI experiments illustrate the development of optic radiations in the left hemisphere, initially not visible at 3 month [7], delineated at 12 month, and clearly reinforced at 20 months of age. The optic radiations recovery in the left hemisphere might occur in congruence with maturation mechanisms observed during the early period of life [34]. Indeed, previous studies have shown that the optic radiation tracts are clearly visible in the newborn (even in premature newborns, e.g. [35]) and their maturation with myelination is largely achieved during the first year of life (e.g. [36]). Here, the differences in visibility with DTI across ages between the optic radiations in left and right hemispheres indicated that recovery mechanisms might be responsible for the development of tracts in the injured hemisphere after perinatal stroke. These arguments support recovered connectivity in the ipsilateral optic radiations that correlated with some of the observed functional responses.

Moreover, the parallel occurrence of optic radiations traced with DTI and the activated visual regions detected with fMRI agreed well with previous findings in healthy adults [37,38]. The correspondence between optic radiations development and visual cortex activation is in line with the observed interdependency between these structures [39]. Thus, the observed functional resilience in this infant agrees well with observations suggesting that plasticity occurs maximally during the first 2 years of age [40]. Different mechanisms underlying plasticity processes have been discussed elsewhere (for review see [41,42]), and include pathway expansion around the area of injury, extending from the retina through the thalamus to extrastriate cortex.

Visual areas showing functional activation were located more laterally in the left hemisphere than in the intact right hemisphere. These more laterally located activation could be part of extrastriate cortex, mainly in V2 area, as defined retinotopically in the adult visual cortex [43,44] and recently shown in children [45]. Although some studies have suggested that activation of the striate cortex was a major sign of visual recovery [46,47], activations limited to extrastriate areas have been reported in patients with brain lesions. Thus, in patients with occipital lobe epilepsy, extrastriate visual responses to full-field visual stimulation were observed with fMRI [48]. In a child with cortical dysplasia fMRI, showed that the activity in the malformed hemisphere was different from that in the intact hemisphere [9], and visual function was related to activations in parieto-temporal regions. In two patients with lesions in striate and prestriate cortex, visual recovery was observed, particularly in the patient with early-onset lesion [49], and correlated to weak fMRI activation of isolated foci in the striate cortex [47]. In addition, it was observed that the stimulation of the scotomatous field in a patient with abnormal striate cortex yielded significant activation in extrastriate areas [50], suggesting important roles of the extrastriate cortex in the process of visual recovery. Recently, it was further shown that after hemianopia, hemianopic hemifield stimulation resulted in bilateral activation restricted to the extrastriate cortex (V2 area) [11]. At 20 months of age it was impossible to carry out clinical visual field testing [51] to determine whether this infant presented a homonymous hemianopic visual field defect. However, the tracts observed in the injured hemisphere and the bilateral cortical activation suggested that both visual hemifields were projected into the visual cortex.

Implication of the injured hemisphere may alternatively be explained by interhemispheric transfer from the intact hemisphere (i.e. transcallosal, major forceps, or commissural tracts) [47,52]. Such interhemispheric connections may play major roles during visual recovery, particularly after early-onset unilateral lesions, as documented previously in animal studies [53]. In our patient, the DTI results showed that the fibers of the optic radiation in the left injured hemisphere concur closely with the functional activation, suggesting a preponderant role for intrahemispheric mechanisms of plasticity. Such recovery (i.e. ipsilesional implication with possible intrahemispheric mechanisms) was also suggested by previous studies in children and adults (e.g. [15,54,55]).

In summary, the fMRI responses concur clearly with the projections of fibers evidenced in DTI maps of both hemispheres. Consequently, it appears that DTI and fMRI findings provide relevant information regarding the recovery of cortical visual processes after early brain lesions, and might contribute to elaborate a pertinent strategy in terms of rehabilitation.

## Abbreviations

DTI Diffusion Tensor Imaging; ER-fMRI Event-Retaled functional Magnetic Resonance Imaging; DTI-12 DTI at 12 months of age; DTI-20 DTI at 20 months of age; FA Fractional Anisotropy.

## Competing interests

The author(s) declare that they have no competing interests.

## Authors' contributions

MLS participated in the acquisition and the analysis of diffusion and functional data and contributed to the interpretation of results and the manuscript redaction. FL participated in the design of the study and the collection of the data and the interpretation of results. SZ participated in the analysis of the diffusion imaging data. SS participated in the design of the study and the monitoring of the sedation of the infant. ABS helped to draft the manuscript. PSH participated in the design and coordination of the study and contributed to the interpretation of results and the manuscript redaction. All authors approved the final manuscript.

## Pre-publication history

The pre-publication history for this paper can be accessed here:


